# Explicative factors of occupational stress among caregivers in hospitals in Brazzaville: a cross-sectional analytical study

**DOI:** 10.11604/pamj.2022.41.197.33309

**Published:** 2022-03-11

**Authors:** Yolande Voumbo Matoumona Mavoungou, Sylvain Honore Woromogo, Levy Mankoussou, Jean Claude Mobousse, Arnold Mangani, Pierre Marie Tebeu

**Affiliations:** 1 Faculty of Health Sciences, University of Marien Ngouabi, Brazzaville, Congo,; 2 Inter State Centre of Higher Education in Public Health for Central Africa (CIESPAC), Brazzaville, Congo,; 3 National Institute of Statistics, Brazzaville, Congo,; 4 Departmental Health Direction, Brazzaville, Congo,; 5 Faculty of Medicine and Biomedical Sciences, University of Yaoundé I, Yaoundé, Cameroon

**Keywords:** Explicative factor, occupational stress, caregivers, Brazzaville

## Abstract

**Introduction:**

staff health represent a population particularly exposed to numerous psycho-social risks. The organization, pace and workload, as well as difficulties in terms of working equipment or personnel have been shown to be a source and consequences of work stress. The objective is to study the factors of stress experienced by health professionals working in district hospitals and to propose preventive actions to decision-makers.

**Methods:**

a cross-sectional analytical study carried out among caregivers in the hospitalization services of Brazzaville. The relationship between stress and certain characteristics of the participants was established using single and multiple logistic regressions taking into account potential confounding factors. For this purpose, Wald Chi-square tests were used as well as the odds ratio with their 95% confidence interval.

**Results:**

midwives have a higher probability of experiencing stress OR = 2.12 [1.13- 4.20] caregivers with less than 10 years of practice are less likely to experience work-related stress OR = 0.53 [0.28 - 0.96] than those with more than 10 years of practice, p = 0.040. After adjusting for possible confounding factors, caregivers who felt useful at work experienced about 5 times more stress odds ratio adjusted (ORa) = 4.69 [1.82 - 12.78] p = 0.002 than those who did not feel useful.

**Conclusion:**

the factors that influence stress among health workers are of a socio-professional nature, and related to the perception of working conditions. Some of them significantly increase the risk of experiencing work-related stress. Further studies are needed to better understand the consequences of occupational stress on the performance of caregivers.

## Introduction

Work stress according to the World Health Organization (WHO) is the set of reactions that employees can have when faced with work demands and pressures that do not correspond to their knowledge and abilities and which question their ability to cope [1,2]. The International Labor Organization indicates that 20 to 25% of workers in developed and developing countries are affected by work-related stress [3-5]. Stressed workers are unable to meet the expectations and demands of their work situation. The consequences for the individual are physical and/or psychological. At the level of work organization absenteeism, the passive presence of the worker can develop over a short, medium and long period of time and lead to a drop in performance and productivity.

Health personnel represent a population particularly exposed to numerous psycho-social risks. The organization, pace and workload, as well as difficulties in terms of working equipment or personnel have been shown to be a source and consequences of work stress. Certain categories of health workers are more vulnerable than others, and the assessment and management of occupational stress are still poorly understood [2,6]. This scourge is also observed in the Congo, where it was noted that particularly in Pointe-Noire, caregivers were at high risk of the negative impact of psychosocial risk factors at work [7]. In Brazzaville, stress among health professionals is poorly documented. The objective is to study the factors of stress experienced by health professionals working in district hospitals and to propose preventive actions to decision-makers.

## Methods

**Design of study:** this is a cross-sectional analytical study carried out during the month of February 2021, among health care personnel in the hospitalization services (medicine, surgery, gynecology and obstetrics) of the four district hospitals of Brazzaville, including Talangaï, Makélékélé, Mfilou and Bacongo.

**Population and sampling:** the study population consisted of nursing staff from the inpatient departments of each of the four hospitals in Brazzaville health districts. All medical, surgical and obstetrical staff who agreed to participate in the study were included. Any staff who were absent during the data collection period were excluded. A comprehensive sample by attendance and membership consisted of the caregivers (doctors, midwives and nurses) working in the study units.

**Data collection:** the data were collected using a self-administered questionnaire from consenting agents in the various departments retained in the study. This questionnaire was pre-tested with health workers working in health department not included in the study. The principal investigator explained the objectives of the study, provided additional explanations to the interviewees and depending on the respondent's availability, the questionnaire was filled on site or completed later by the interviewee alone and given to the interviewer.

**Variables:** the dependent variable of the study is the perception of stress by the agents surveyed. The explicative variables considered are the socio-demographic and professional characteristics and the characteristics related to the perception of the working environment conditions. Socio-demographic and professional characteristics were age, gender, marital status, number of dependents; qualification, having heard about stress, number of years in service and status in the department. The characteristics related to the perception of workplace conditions were the perception of stress, usefulness in the department, job description, workload, participation in work organization, and availability of work materials, professional relations with colleagues or superiors.

**Data analysis:** these data were entered with the Cspro version 7.4 software and analyzed with the software R version 3.6.1. The results were presented as an average, as a proportion. The relationship between stress and certain characteristics of the participants was established using single and multiple logistic regressions taking into account potential confounding factors. For this purpose, Wald Chi-square tests were used as well as the odds ratio with their 95% confidence interval. The difference was significant if p <0.05. Multivariate logistic regression with stepwise elimination with p <0.20 was used to predict caregiver stress

**Ethical considerations:** the authorization of the Departmental Director of Health and that of the heads of hospitals in Brazzaville health districts were required. Interviewees were made aware of the voluntary nature of participating in the study. Their verbal consent was obtained after presentation of the study objectives, the data collected was anonymous; respondents were free to withdraw from the study.

## Results

Among the 420 eligible caregivers in the targeted services, 353 agreed to participate in the study, with a participation rate of 84.04%. The predominance of female professionals (84.4%) was noted, and the F/H sex ratio of the sample was estimated at 3.62. The socio-professional characteristics of the respondents are presented in Table 1.

**Table 1:** distribution of respondents by socio-professional characteristics (N=353)

Variables	Number	Percentage (%)
**Sex**		
Female	295	84.40
Male	55	15.60
**Age (years)**		
< 30	107	30.30
30 - 40	114	32.30
40 - 50	94	26.60
> 50	38	10.80
**Marital status**		
As a couple	137	38.80
Lives alone	216	61.20
**Number of dependents**		
No charge	81	22.95
1 - 3 people	141	41.64
>3	125	35.41
**Status in the service**		
Official	141	39.90
Non-public servant	212	60.10
**Qualification**		
Doctor	39	11.05
Nurse	195	55.24
Midwife	119	33.71
**Have heard of work stress**		
Yes	321	90.93
No	32	09.07
**Number of years in practice**		
< 10	215	60.91
> 10	138	39.09

**The distribution of caregivers according to the perception of the characteristics of the work environment:** the distribution of caregivers according to perceived workplace characteristics is presented in Table 2. The proportion of caregivers in the sample who reported feeling stress was 291 (82.44%). The majority of the caregivers surveyed (92.35%) reported that their job description was appropriate to their knowledge. The workload was considered normal by 86.97% of the caregivers. For more than half of the respondents, professional relations were considered unsatisfactory both with colleagues (56.09%) and with superiors (54.95%).

**Table 2:** distribution of caregivers by perception of working conditions

Variables	Number	Frequency (%)
**Stress felt**		
Yes	291	82.44
No	62	17.56
**Feels useful or valued in the service**		
Yes	247	69.97
No	106	30.03
**Description of your tasks**		
Adapted to your knowledge	326	92.35
Not adapted	27	07.65
**Satisfaction of working conditions**		
Yes	54	15.30
No	299	84.70
**Participation in the organization of the department's activities**		
Never	146	3641
Sometimes	141	39.94
Always	66	18.70
**Availability of the necessary working material**		
Never	64	18.13
Sometimes	256	72.52
Always	33	09.35
**Perception of workload**		
Low	19	05.38
Normal	307	86.97
High	27	07.65
**Professional relations with colleagues**		
Unsatisfactory	198	56.09
Low satisfaction	98	27.76
Satisfactory	57	16.15
**Professional relations with hierarchical superiors**		
Unsatisfactory	194	54.95
Low satisfaction	92	26.06
Satisfactory	67	18.98

**The influence of socio-occupational factors and the perception of the characteristics of the work environment on caregivers experiencing stress:** midwives (33.71%) have a higher probability of experiencing stress OR = 2.12 [1.13 - 4.20] with a significant difference p = 0.024. Caregivers with less than 10 years of practice (39.9%) are less likely to experience work-related stress OR = 0.53 [0.28 - 0.96] than those with more than 10 years of practice (60.9%), the difference is statistically significant p = 0.040. Those who have heard about work-related stress (90.93%) are 13 times more likely to experience it OR = 13.04 [5.97 - 29.96]; the statistical difference is significant p = 0.004 (Table 3). However, stress has no influence on the gender, age and status of caregivers.

**Table 3:** influence of socio-professional characteristics on perceived stress

Variables	Stress			
	Yes (n = 291)	No (n = 62)	OR (95 % CI)	p
**Sex**				
Female	246	52	1.05 (0.49 - 2.22)	0.438
Male	45	10	1	
**Age group**				
< 30	87	20	1	
30 - 40	93	21	1.01 (0.51 - 2.01)	0.547
40 - 50	79	15	1.21 (0.58 - 2.52)	0.347
> 50	32	06	1.22 (0.45 - 3.32)	0.448
**Marital status**				
As a couple	109	28	1	
Alone	182	34	1.37 (0.79 - 2.39)	0.161
**Number of dependent children**				
No charge	63	18	1	
1 - 3	123	24	1.46 (0.74 - 2.89)	0.178
> 3	105	20	1.50 (0.74 - 3.05)	0.173
**Salary status**				
Official non-public	119	22	1.26 (0.71 - 2.22)	0.260
Servant	172	40	1	
**Qualification**				
Nurses	152	43	1	
Doctors	34	05	1.92 (0.71 - 5.22)	0.137
Midwives	105	14	2.12 (1.10 - 4.07)	0.014
**Number of years in practice**				
>10	121	17	1.88 (1.03 - 3.45)	0.025
<10	170	45	1	
**Have heard of stress**				
Yes	280	41	1	
No	11	21	0.08 (0.03 - 0.17)	< 10-3

**Perception of workplace characteristics that affect caregivers experiencing stress:** most caregivers feel useful at work (69.97%) their probability of feeling stress at work is three times higher OR = 3.13 [1.78 - 5.52] than those who do not feel useful; the observed difference is statistically significant p = 0.001 (Table 4). After adjusting for possible confounding factors, caregivers who felt useful at work experienced about 5 times more stress ORa = 4.69 [1.82 - 12.78] p = 0.002 than those who did not feel useful. Caregivers who felt less stress at work were those with less than 10 years of service ORa = 0.33 [0.13 - 0.81] p = 0.018; those who reported that they always had work equipment ORa = 0.04 [0.01 - 0.19] p = 0.001, as well as those who reported having satisfactory relations with superiors OR = 0.01 [0.00 - 0.04] p = 0.001 and colleagues ORa = 0.11 [0.04 - 0.028] p = 0.001 (Table 5). However, caregivers who are satisfied with their working conditions (15.30%) are less likely to experience work-related stress OR = 0.55 [0.28 - 1.11] the difference is not significant p>0.05.

**Table 4:** influence of the perception of working conditions on the stress experienced by caregivers

Variables	Stress			
	Yes (n = 291)	No (n = 62)	OR (95 % CI)	p
**Satisfaction of working conditions**				
Yes	40	14	0.55 (0.28 - 1.08)	0.063
No	251	48	1	
**Feels useful or valued in the work**				
Yes	217	30	3.12 (1.78 - 5.49)	< 10-3
No	74	32	1	
**Description of tasks**				
Knowledge-adapted	274	52	3.09 (1.34 - 7.15)	0.009
Not adapted	17	10	1	
**Workload perception**				
Normal	257	50	1	
Low	13	06	0.42 (0.15 - 1.16)	0.086
High	21	06	0.68 (0.26 - 1.77)	0.288
**Participation in the organization of the department's activities**				
Never	133	13	1	
Rarely	113	28	0.39 (0.19 - 0.79)	0.006
Always	45	21	0.21 (0.09 - 0.45)	< 10-3
**Availability of work material**				
Never	59	05	1	
Sometimes	216	40	0.46 (0.17 - 1.21)	0.074
Always	16	17	0.08 (0.03 - 0.25)	< 10-3
**Working relationships with colleagues**				
Unsatisfactory	190	08	12.69 (5.82 - 27.72)	< 10-3
Satisfactory	101	54	1	
**Working relationship with hierarchical superiors**				
Unsatisfactory	191	03	37.56 (11.49 - 122.85)	< 10-3
Satisfactory	100	59	1	

**Table 5:** factors associated with the stress experienced by caregivers in Brazzaville

Variables	Stress					
	Yes	No	OR (95 % CI)	p	ORa (95 % CI)	p
**Qualification**						
Nurse and others	152	43	0.51 (0.19 - 1.41)	0.137		
Doctor	34	05	1			
Midwife	105	14	1.10 (0.37 - 3.28)	0.527		
**Number of years in practice**						
> 10	121	17	1.88 (1.03 - 3.45)	0.025	1.67 (1.07 - 2.55)	0.018
< 10	170	45	1			
**Feels useful or valued at work**						
Yes	217	30	3.13 (1.78 - 5.79)	< 10-3	4.69 (1.82 - 121.78)	0.002
No	74	32	1		1	
**Perception of job descriptions**						
Adapted to your knowledge	274	52	1			
Not adapted	17	10	0.32 (0.14 - 0.74)	0.009	0.57 (0.19 - 1.76)	0.322
**Availability of work equipment**						
Never	59	05	12.53 (4.01 - 39.20)	< 10-3	3.05 (2.99 - 3.99)	0.013
Sometimes	216	40	5.73 (2.68 - 12.29)	< 10-3	2.69 (1.89 - 3.02)	0.001
Always	16	17	1			
**Working relationship with hierarchical superiors**						
Unsatisfactory	191	03	37.56 (11.49 - 122.85)		7.02 (5.66 - 8.21)	0.001
Satisfactory	100	59	1		1	
**Working relationships with colleagues**						
Unsatisfactory	190	08	12.69 (5.82 - 27.72)	< 10-3	4.23 (3.45 - 5.02)	0.001
Satisfactory	101	54	1			

## Discussion

This study is a contribution to the prevention of psychosocial risks in hospitals in Brazzaville. The participation of only those professionals who were present and in good health may constitute a selection bias, since health workers who were off sick due to emotional health problems linked to their work were not taken into account. In addition, the fear of possible reprimands from superiors could influence the quality of the respondents' statements. The participation rate of caregivers was 84.04%. This rate is lower than the one observed by Gounongbé *et al*. which is 96.6% among caregivers at the Mother and Child University Hospital in Cotonou [8]. In our study, this may reflect the existence of a climate of mistrust within the departments and justify the refusal of some caregivers to express themselves about their experiences in the department for fear of reprimands from their colleagues or hierarchical superiors. The predominance of women in the sample (sex ratio F/H = 3.62) for doctors and nurses is in line with the findings of other authors who have shown that the feminization of the medical profession is a growing phenomenon [9,10]. In our study, this could also be justified by taking into account the maternity ward, which also has the highest frequency of midwives in the sample. The mean age in our sample was 37.30 +/- 9.68 years. Some authors have shown an average age slightly higher than ours [7,8]. This difference can be explained by the fact that almost 40% of the staff in the 4 district hospitals in Brazzaville have been in the service for more than 10 years. It is important to strengthen the staff in these hospitals in terms of numbers.

**Perception of workplace characteristics:** the proportion of caregivers in the sample who reported feeling stress was 82.44%. This proportion of occupational stress is higher than that observed by some authors [10,11]. The data from our study corroborate, however, with the finding of the WHO in 2006 that the stress situation in health facilities in many sub-Saharan African countries is critical [3] and another study in the United States where it is reported that three-quarters of Americans suffer from stress-related symptoms in a given month [12]. This work draws the attention of health district hospital managers to the problem of occupational stress among health care workers and suggests that further studies be carried out on the repercussions of this on their performance within the health services, in order to implement appropriate preventive measures. Most of the caregivers (92.35%) stated that their job description was adapted to their knowledge. The proportion of those who stated that they were never involved in the organization of activities on the ward was 41.36%. The involvement of caregivers in the organization of activities is likely to create a sense of belonging, an awareness of their role in the provision of care. In our context, this suggests that most often caregivers are involved in a clinical governance process that promotes effective collaboration between caregivers in the delivery of activities within the service.

About three quarters (75.35%) of the respondents stated that working materials are sometimes available. The irregularity of the care materials declared by the caregivers in our study is in line with the observation made by Bara *et al*. [13] who noted the frequent shortage of medicines and medical consumables in Burkina Faso. The workload is considered normal by 86.97% of the caregivers. An individualized approach to the workload is necessary to better identify. Professional relations are considered unsatisfactory both with colleagues (56.09%) and with superiors (54.95%); these data corroborate those revealed in a study that there are conflictual relations at work [14].

In our study, the influence of variables such as gender, age, and professional status of caregivers who experience work-related stress is not statistically significant p>0.05. These data are contrary to those reported by some authors [5] who have shown that stress is higher in women, young people under 40 years of age and people in the 45-54 age group are more concerned. Caregivers with less than 10 years of service are less likely to feel stressed at work. This result is contrary to the findings of other authors [15,16]. In our study, this suggests that despite the increase in the number of years of seniority of the caregiver, he or she may have difficulty developing mechanisms to overcome certain constraints of the work environment. Moreover, the fact that many (60.9%) remain in a non-civil servant salary status after more than 10 years of practice could also induce stress. Stress prevention measures should be promoted and an appropriate working environment should be ensured to enable caregivers to combat psychosocial risks. Midwives are twice as likely to experience work-related stress as other caregivers, which is consistent with the finding of Creedy *et al*. [17]. However, this situation, which was not confirmed in a multivariate analysis, suggests that further studies should be carried out on occupational stress in this category of caregivers, and that effective measures for the prevention of occupational stress should be implemented in this care environment.

Most of the caregivers surveyed felt less stress when the availability of the work tool increased, with a statistically significant difference. This result is in line with those of other authors [18,19] who report that poor working conditions are a cause of occupational stress among caregivers. Caregivers who reported satisfactory working relationships with both colleagues (16.15%) and supervisors (18.98%) felt less stress than their colleagues; the difference found was statistically significant. This implies that a high proportion of caregivers report unsatisfactory relationships, and a higher probability of feeling stress. This result is consistent with other authors who find that unsatisfactory relationships are a source of stress at work [6,17,20]. In our study, this situation could be explained by communication problems and a lack of understanding of the tasks and roles of caregivers within the work teams. It is likely to lead to a deterioration in the working atmosphere, to have repercussions on the health of the caregivers but also on the quality of care administered to patients, as reported by [17]. Therefore, the carrying out of a regular analysis of working conditions aimed at identifying stressful situations with a view to correcting the difficulties is a hypothesis to be developed.

## Conclusion

More than three quarters of the care workers in the maternity, medicine and surgery departments of hospitals in the health districts of Brazzaville say they feel stress at work. The factors that influence stress among health workers are of a socio-professional nature, and related to the perception of working conditions. Some of them significantly increase the risk of experiencing work-related stress. Further studies are needed to better understand the consequences of occupational stress on the performance of caregivers. Raising awareness of psychosocial risks, training and supervision of caregivers on the job and improving working conditions are likely to prevent the occurrence of stress among caregivers.

### What is known about this topic

- Caregivers with less than 10 years of practice are less likely to experience work-related stress than those with more than 10 years of practice;
- Midwives and others have a higher probability of experiencing stress than doctors;
- Heard about stress at work helps to reduce stress.

### What this study adds

- Unsatisfactory working relationships with colleagues increases the risk of stress for caregivers;
- Availability of work equipment reduces stress;
- Raising awareness of psychosocial risks, training and supervision of caregivers on the job and improving working conditions are likely to prevent the occurrence of stress among caregivers.
